# The Content of Imagined Sounds Changes Visual Motion Perception in the Cross-Bounce Illusion

**DOI:** 10.1038/srep40123

**Published:** 2017-01-10

**Authors:** Christopher C. Berger, H. Henrik Ehrsson

**Affiliations:** 1 Department of Neuroscience, Karolinska Institutet, Retzius väg 8, SE-171 77 Stockholm, Sweden.

## Abstract

Can *what* we imagine hearing change what we see? Whether imagined sensory stimuli are integrated with external sensory stimuli to shape our perception of the world has only recently begun to come under scrutiny. Here, we made use of the cross-bounce illusion in which an auditory stimulus presented at the moment two passing objects meet promotes the perception that the objects bounce off rather than cross by one another to examine whether the content of imagined sound changes visual motion perception in a manner that is consistent with multisensory integration. The results from this study revealed that auditory imagery of a sound with acoustic properties typical of a collision (i.e., damped sound) promoted the bounce-percept, but auditory imagery of the same sound played backwards (i.e., ramped sound) did not. Moreover, the vividness of the participants’ auditory imagery predicted the strength of this imagery-induced illusion. In a separate experiment, we ruled out the possibility that changes in attention (i.e., sensitivity index *d*′) or response bias (response bias index *c*) were sufficient to explain this effect. Together, these findings suggest that this imagery-induced multisensory illusion reflects the successful integration of real and imagined cross-modal sensory stimuli, and more generally, that what we imagine hearing can change what we see.

We usually believe that the things we imagine in our mind and the things we perceive in the external world are perceptually distinct from one another; and therefore, that what we imagine should not influence what we perceive. However, decades of research on mental imagery—one’s willed simulation of sensory perception—has found that this is not always the case^1^,^2^,^3^,^4^ and that imagined and real sensory stimuli often reflect similar processes, both phenomenologically and in the brain^5^,^6^,^7^,^8^,^9^,^10^,^11^,^12^,^13^,^14^. Classic studies on auditory imagery, for instance, have found that imagining a tone can aid the detection of that tone^3^ and that the time required to scan an imagined familiar song for a specific lyric (a task that requires auditory imagery) is proportional to the time it takes for that lyric to occur in the song^6^. Furthermore, neuroimaging studies have found that imagining a sound activates secondary auditory areas^15^,^16^,^17^ and that imagining sounds of different frequencies activates frequency-specific areas of the auditory cortex^13^.

Perception, however, is largely multisensory, and a coherent representation of the world around us involves the successful integration of our different senses^18^,^19^. Despite the abundance of research highlighting the similarities between imagery and perception within a single sensory modality, the question of whether mental imagery can also integrate with stimuli from a different sensory modality to change our perception of the world is only beginning to be examined^20^,^21^. If auditory imagery and auditory perception indeed involve overlapping representations, then we should expect imagined auditory stimuli to interact with real visual stimuli in the same manner that real auditory stimuli interact with real visual stimuli. Multisensory illusions provide a useful tool with which to investigate this possibility.

Studies on the classic cross-bounce illusion—a multisensory illusion in which a sound changes the perceived motion of two moving objects^22^—have found that two passing discs are perceived as bouncing off one another rather than crossing one another when the sound is presented near or at the moment two moving discs meet^22^,^23^,^24^. Further experiments on this classic illusion have successfully ruled out alternative explanations for the illusion, such as attention or response bias, by manipulating the kind of sound presented and the extent to which the discs overlap when they meet at the center of the screen. For example, Grassi and Casco^25^ found that an arbitrary synthetic tone that had been damped and thereby simulated the typical acoustics of colliding objects increased the bounce-percept, whereas the same tone played in reverse (i.e., ramped) did not (even though the ramped control stimulus was perceived as more perceptually salient than the damped stimulus). This finding is consistent with the ‘unity-assumption’ principle of multisensory integration, which states that only meaningful combinations of cross-modal sensory stimuli are integrated^26^,^27^,^28^. Furthermore, because these two sound conditions are matched in terms of their influence over executive attention, Grassi and Casco^25^ concluded that this multisensory illusion cannot be explained by changes in attention alone. Additionally, consistent with the fact that truly colliding objects do not overlap (but passing objects do), Grassi and Casco^25^ also found that the bounce-percept increased when the participants heard the damped sound at the moment the discs met when the overlap of the discs was decreased. The extent to which the stimuli overlap decreases the probability that two objects are perceived as colliding because colliding objects generally do not overlap at all, whereas passing objects do (in this way, although it has become conventional to describe the perceived motion of the discs on fully overlapping trials as ‘bouncing,’ the term ‘bounce’ is not entirely accurate since truly bouncing objects never overlap). The observation that the increased probability of perceiving a bounce-percept when hearing a damped sound scaled according to the decrease in the amount that the discs overlapped suggested that a general response-bias heuristic related to a particular sound condition (i.e., one hears the damped sound and always presses bounce) is also not sufficient to explain the effect^25^,^29^. In this way, the cross-bounce illusion is a particularity suitable paradigm to investigate how auditory imagery influences multisensory perception while simultaneously ruling out attention- or response bias-based accounts. Thus, we sought to extend recent findings that demonstrate that imagined auditory stimuli can change visual motion perception in the cross-bounce illusion^20^ to examine whether this change in perception follows the same unity assumption principle of multisensory integration as the integration of real cross-modal stimuli.

## Experiments 1a and 1b

The aim of Experiment 1a was to examine whether imagining a sound *per se* or the content of the imagined sound would induce the cross-bounce illusion. Consistent with findings on the perceptual version of the cross-bounce illusion^25^,^29^, we predicted that imagining a damped sound at the moment the discs met would increase the proportion of the perceived bounce trials compared to imagining ramped and no-sound conditions. To ensure that any observed differences between the imagery conditions (i.e., damped sound imagery, ramped sound imagery, no sound imagery) could not be explained by a general response strategy, we also manipulated the extent to which the discs overlapped at the center of the screen before continuing in their original trajectory (100%, 80%, and 60% overlap). Thus, we predicted that if the tendency to report that the discs bounce in the damped sound condition was merely a response bias rather than perceptual in nature, the proportion of reported bounce trials would be consistent across the overlap conditions. However, if the participants were making trial-by-trial judgments about their perception in the damped sound condition, then we predicted that the proportion of reported bounce trials would increase as a function of the extent to which the discs did not fully overlap. This prediction is in accordance with previous studies on the cross-bounce effect described above^25^,^29^. The aim of Experiment 1b was to examine whether a real sound produced the same results as Experiment 1a; thus, the sounds were played aloud rather than imagined at the moment the discs met.

## Experiment 2

The purpose of Experiment 2 was to directly examine whether there were any changes in visual attention or response-bias tendencies related to the imagine damped, imagine ramped, and no sound imagery conditions that could account for any observed results in Experiment 1a. To this end, we tested whether the imagined sounds affected the participant’s ability to discriminate whether the discs overlapped at the center of the screen and whether the imagined sounds changed the participants’ response tendencies. Directly in accordance with previous studies on the cross-bounce illusion described above^25^,^29^, we predicted that if the act of imagining a sound detracts or enhances the participants’ visual attention, then we would observe a difference in the discrimination of the disc overlap across the different imagery conditions; similarly, a systematic response-bias would reveal itself in the form of a significant difference in the response tendencies between imagery conditions. In contrast, no difference in performance on a visual discrimination task or in the response tendencies between the two imagined sound conditions during the visual discrimination task would provide further support for a multisensory integration account of any observed differences in the proportion of perceived bounce for imagined damped sounds in Experiment 1a.

## Methods

### Participants

In total, seventy-two healthy participants participated in this study. Twenty-four participants (mean age = 27.6 years, *SD* = 6.2; 14 females) participated in Experiment 1a, twenty-four participants (mean age = 24.5 years, *SD* = 3.7; 18 females) participated in Experiment 1b, and twenty-four (mean age = 26.1 years, *SD* = 5.7; 12 females) participated in Experiment 2. None of the participants participated in more than one experiment. All participants were recruited from the student population in the Stockholm area, were healthy, and reported no history of psychiatric illness or neurologic disorders and reported no problems with hearing or vision (or had corrected-to-normal vision). A sample size of 22 participants was predetermined to yield a power of 80% and to detect an effect of comparable size to previous studies using similar independent and dependent measures^20^,^21^,^30^,^31^; thus, data collection was stopped once the appropriate number of participants was reached (after any exclusions), and the experiment was fully counterbalanced. The number of participants recruited for Experiments 1b and Experiment 2 was determined to match the final number of participants from Experiment 1a. In addition to the 24 participants who were included in each experiment, one additional participant was excluded from Experiment 1a and two from Experiment 1b for problems with their vision, and one additional participant was excluded from Experiment 1a because they did not understand the task. These additional participants did not complete all blocks of the experiment; data collection ceased once the contradiction was made known to the experimenter following the first block of trials. All participants provided written informed consent before the start of the experiment, and the experiments were approved by and conducted in accordance with the ethical guidelines of the Regional Ethical Review board of Stockholm.

### Experiments 1a and 1b

#### **Stimuli**

**Figure 1:**
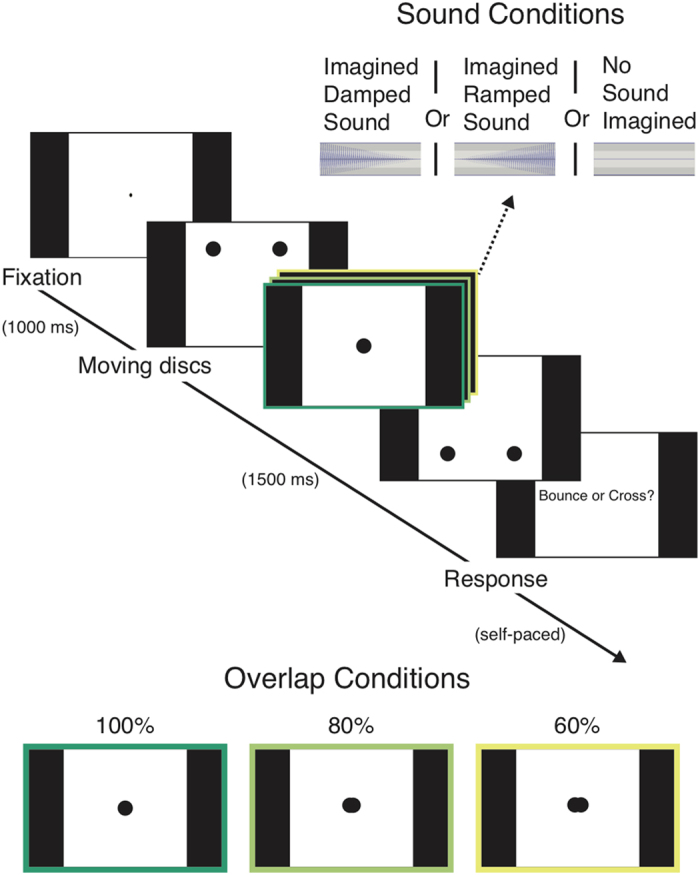
Schematic overview of the experimental setup and the different overlap conditions used in all experiments and the imagined sound conditions used in Experiment 1a and Experiment 2. In Experiment 1b, the sounds were played to the participants, rather than being imagined, at the moment the discs met. In Experiment 2, the Response question asked whether the discs partially overlapped or completely overlapped rather than whether the discs bounced or crossed. For display purposes, the sizes of the discs and the percentage overlap conditions are not drawn exactly to scale.

The visual stimuli for all the experiments consisted of two black discs (diameter = 0.702°) descending at a 45° angle from the top left and right corners of a white square (height = 15.095°, width = 15.095°) and finishing in the bottom right and left corners of the screen, respectively (Fig. 1). The discs either overlapped completely (100% overlap) at the center of the screen, overlapped by 80% at the center of the screen, or overlapped by 60% at the center of the screen (Fig. 1). The moving discs for all overlap conditions had the same trajectory; however, the 80% and 60% overlap trials were achieved by removing the frames (0.561° for the 60% overlap condition; 0.281° for the 80% overlap condition) for which the overlap of the discs’ diameters (in the horizontal plane) was greater than 80% and 60%, respectively (see Supplemental Media for examples). To keep the total duration (1500 ms) and speed (10.081°/s) of the discs constant in all three overlap conditions, the frames removed from the overlap of the discs in the 60% and 80% overlap conditions were added to the start and end points of each disk. Thus, the discs started and finished further ‘off-screen’ in the 60% and 80% overlap conditions than in the 100% overlap condition.

The damped auditory stimulus that participants were instructed to imagine in Experiment 1a was a 200 ms harmonic complex tone that consisted of the sum of harmonics 1–10 of a 250 Hz fundamental for which the maximum amplitude was linearly decreased from ± 1 to ± 0 across its duration. The ramped auditory stimulus was the same as the damped auditory stimulus but was played backwards (Fig. 1; see also Supplemental Media).

In Experiment 1b, in which the ramped and damped sounds were actually played (rather than imagined), the sounds were triggered at the moment the discs met onscreen, i.e., 0.702° before the discs completely overlapped (or would have completely overlapped in the case of the 80% and 60% overlap conditions). The ramped and damped sounds were played to the participants with a maximum recorded volume of 60 dBA.

For all experiments, the stimuli were presented and controlled using PsychoPy^32^,^33^ on a 21.5 inch (1920 × 1080 p) iMac (OS X Mavericks, Version 10.9.5) computer (refresh rate = 60 Hz) at a viewing distance of 100 cm in a soundproofed testing room, and all analyses and statistical tests were performed using the statistical software R^34^.

#### **Procedure**

Prior to the start of the experiment, the participants were informed that they would see two discs moving diagonally across the screen. They were also shown a visual depiction of what would appear on the screen and were informed that there were two possible ways to perceive the discs’ motion, i.e., that the discs can be seen as bouncing off or crossing one another. They were then informed that their primary task was to report whether they saw the discs bounce off or cross one another. Next, the participants were also informed that in two of the blocks of the experiment, they would be asked to imagine hearing a specific sound during each trial and that the experimenter would play that sound for them several times before the start of that block so they could have a good mental representation of it before the start of that block. To minimize confusion about the task and to ensure that the participants were able to distinguish between the two sounds well enough to imagine them, both sounds were played once prior to the start of the experiment, and the participant was asked whether they could recognize any difference between them. All participants recognized that the sounds were different. Then, in three separate blocks, the participants were either instructed to imagine hearing the damped sound at the moment the disks met (i.e., 0.702° before 100% overlap; see Stimuli section above for details), imagine hearing the ramped sound at the moment the discs met, or to simply observe the visual stimuli. The order of the imagery blocks was counterbalanced across participants, and the no sound imagery block always appeared between the sound imagery blocks to allow sufficient time between each imagery condition for the participants to keep a clear and correct auditory image in mind. Prior to each imagery block, the ramped or damped sound was played several times (≈10 times) for the participant until they felt they had a clear memory of the stimulus. Each block of trials consisted of twenty-five 100% overlap trials, twenty-five 80% overlap trials and twenty-five 60% overlap trials (225 trials in total). At the conclusion of the experiment, the participants were given a questionnaire that asked them to rate, on a scale from 1–10, how vividly (1 being not at all, and 10 being extremely well and vividly) they were able to imagine hearing the sounds during the experiment. All procedural details for Experiment 1b were identical to those of Experiment 1a, except that the participants were not played the sound several times before the ramped and damped sound blocks, the sounds were actually played to the participants during the trials of those blocks, and the participants were instructed to listen to the sounds rather than imagine hearing them.

### Experiment 2

All stimuli and procedures for Experiment 2 were identical to those of Experiment 1a with the following exception: Rather than informing participants that there were two possible ways of seeing the discs move (i.e., to bounce or cross), the participants were instead informed that the discs would either fully overlap in the center or would only partially overlap and that their primary task was to determine whether they saw the discs partially overlap or fully overlap in each trial. Thus, the question following each trial asked participants whether the discs partially overlapped or fully overlapped rather than whether they saw the discs bounce or cross.

## Results

### Experiment 1a

**Figure 2:**
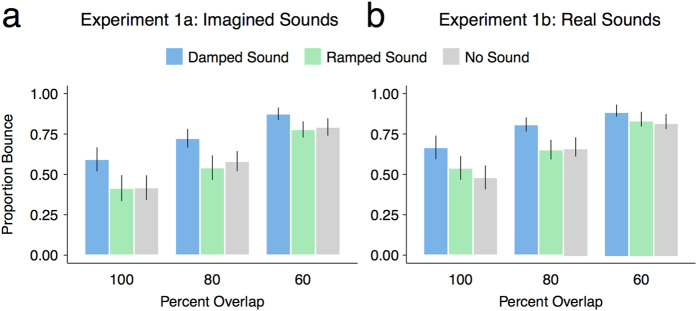
Mean proportion of perceived bounce as a function of the percent overlap of the moving discs and the kind of imagined sound in Experiment 1a (**a**) and heard sound in Experiment 1b (**b**). The error bars represent ± SE.

In this experiment, we examined whether the imagery-induced cross-bounce illusion (i.e., increase in the proportion of perceived bounce from imagined auditory stimuli) adhered to the unity assumption hypothesis of multisensory integration by manipulating the content of the imagined sounds. Specifically, we were interested in examining whether imagining a damped sound significantly increased the probability of a bounce percept compared to imagining a ramped sound or not imagining a sound at the moment two passing discs met. Additionally, we also examined whether the increase in the perceived bounce scaled according to the extent to which the discs did not overlap, which would demonstrate that the participants were responding on the basis of their genuine perception rather than according to a response bias (i.e., with approximate equal frequency across the overlap conditions). Thus, in order to examine whether the imagined sounds and the percent overlap affected the proportion of perceived bounce, a generalized linear mixed model (GLMM) was fit to the binomial data (using a logistic link function) with sound condition and percent overlap (mean centered) as fixed factors and by-participant random intercepts and slopes of the effects of sound condition and percent overlap^35^. This analysis was performed using the lme4 package^36^ in the R environment^34^ and allowed us to examine the effect of sound condition and percent overlap manipulations while controlling for individual variability. This analysis revealed that the imagined damped sound significantly increased the probability of a bounce percept compared to the no-sound condition (*β* = 1.49, *SE* = 0.48, *z* = 3.12, *p* = 0.002), but that the imagined ramped sound did not (*β* = −0.05, *SE* = 0.50, *z* = 0.06, *p* = 0.98). This analysis also revealed a significant negative relationship between the extent that the discs overlapped and the probability of a bounce percept (*β* = −0.06, *SE* = 0.01, *z* = −4.55, *p* < 0.001). In order to assess weather there was a significant difference between the effects of the damped- and ramped-sound conditions on the perception of the bounce percept the above model was re-run with the ramped-sound condition set as the reference for the fixed factor sound condition. This analysis revealed that the damped sound significantly increased the probability of a bounce percept compared to the ramped sound (*β* = 1.51, *SE* = 0.61, *z* = 2.45, *p* = 0.01). The proportions of reported bounce trials were calculated for each overlap condition for each imagery condition (no-imagery, imagine damped sound, imagine ramped sound) separately and are reported in Fig. 2a.

In order to assess whether the increase in the bounce percept in the damped sound condition was consistent with a genuine change in perception rather than a general response strategy, we also performed Wilcoxon signed-rank tests between the 60% vs. 80%, and 80% vs. 100% overlap conditions to ensure that the bounce percept decreased as a function of the extent that the discs overlapped for this condition. We found significant differences between the 60% and 80% overlap conditions (*z* = 2.95, *p* *=* 0.002) and between the 80% and 100% overlap conditions (*z* = 3.36, *p* < 0.001). This analysis ensured that the increase in the probability of a bounce percept for the damped-sound condition in the GLMM analysis above could not be explained by a general response bias, suggesting that the participants were making trial-by-trial judgments about their perception.

**Figure 3:**
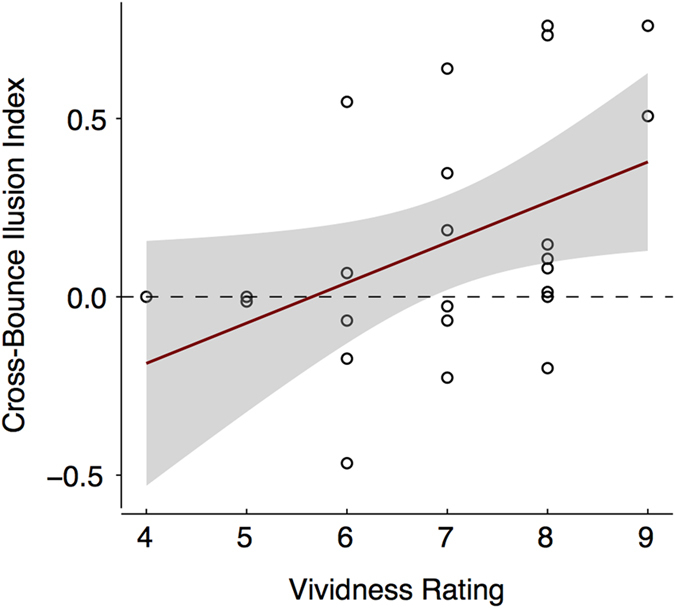
Regression plot (and 95% confidence bands) showing the relationship between the participants’ self-reported vividness of the imagined sounds and the strength of the cross-bounce illusion. The dotted line denotes the divide between the participants who perceived the discs to bounce more when they imagined the damped compared to ramped sound and the participants who did not.

We also investigated whether there was any relationship between the participants’ self-reported vividness of the imagined sounds and the strength of the imagery induced cross-modal effect. For this analysis, an imagery-induced cross-bounce illusion index was calculated for each participant. The cross-bounce illusion index was calculated as the mean of the differences between the proportion of perceived bounce when the participants imagined hearing the damped sound and the proportion of perceived bounce when they imagined hearing the ramped sound across the three overlap conditions. A key feature of this estimate is that it reflects the strength of the imagery-induced illusion while controlling for any effect that could be explainable by imagining the sound *per se* (e.g., attention or response bias). We found a significant positive relationship [*F*(1, 22) = 4.93, *p* = 0.037, *R*^2^ = 0.18] between the strength of their cross-bounce illusion and the strength of their self-reported ability to imagine the sounds (Fig. 3). That is, the more vividly the participants were able to imagine hearing the sounds, the stronger their imagery-induced cross-bounce illusion.

### Experiment 1b

In this experiment we wanted to confirm that the presentation of real sounds affected the participants’ perception of the moving discs in the same manner as the imagined sounds (as described above). To this end, we used the same paradigm and analyses as in Experiment 1a except that we presented actual sounds instead of instructing the participants to imagine the sounds. In line with the previous findings^25^,^29^, we expected the damped sound to significantly increase the probability of a bounce percept compared to when the ramped sound was presented or no sound was presented at the moment the two passing discs met. We also expected that any increase in the perceived bounce should scale according to the extent to which the discs did not overlap^29^. To test these predictions a GLMM was once again fit to the binomial data (using a logistic link function) with sound condition and percent overlap (mean centered) as fixed factors and by-participant random intercepts and slopes of the effects of sound condition and percent overlap. This analysis revealed that the imagined damped sound significantly increased the probability of a bounce percept compared to the no-sound condition (*β* = 1.13, *SE* = 0.42, *z* = 2.66, *p* = 0.008), but that the imagined ramped sound did not (*β* = 0.03, *SE* = 0.29, *z* = 0.09, *p* = 0.926). This analysis also revealed a significant negative relationship between the extent that the discs overlapped and the probability of a bounce percept (*β* = −0.052, *SE* = 0.01, *z* = −3.71, *p* < 0.001). In order to assess weather there was a significant difference between the effects of the damped- and ramped-sound conditions on the perception of the bounce percept the above model was re-run with the ramped-sound condition set as the reference for the fixed factor sound condition. This analysis revealed that the damped sound significantly increased the probability of a bounce percept compared to the ramped sound (*β* = 1.10, *SE* = 0.39, *z* = 2.84, *p* = 0.004).

Also consistent with the findings from Experiment 1a, planned comparisons confirmed that the proportion of perceived bounce trials when participants heard the damped sound was significantly greater for the 60% overlap compared to the 80% overlap trials (*z* = 2.10, *p* = 0.036), and was significantly greater for the 80% overlap trials compared to the 100% overlap trials (*z* = 2.95, *p* = 0.022).

### Experiment 2

**Figure 4:**
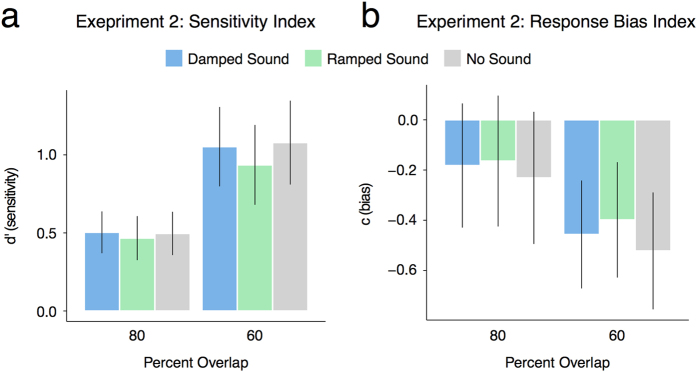
Mean sensitivity index *d*′ (**a**) and response bias index *c* (**b**) as a function of the percent the moving discs that overlapped in the two partial overlap conditions and the kind of imagined sound in Experiment 2. The error bars represent ± SE.

In this experiment we wanted to rule out the possibility that imagining the different sounds (i.e., damped or ramped) could result in changes in visual attention or response strategies that could account for the changes in perception we observed in Experiment 1a. To address this question, we transformed the experimental paradigm into a visual detection task by asking the participants to report whether they perceived the discs to fully overlap or not^25^ while keeping all other procedures and materials identical to Experiment 1a. Thus, in order to assess whether there were any changes in visual attention or response-bias tendencies related to the imagine damped, imagine ramped, and no sound imagery conditions that could account for any observed results in Experiment 1a, the participants’ responses were coded as hits (i.e., a partially overlapping response was given for a partially overlapping display), misses (i.e., a fully overlapping response was given for a partially overlapping display), correct rejections (i.e., a fully overlapping response was given for a fully overlapping display), and false alarms (a partially overlapping response was given for a fully overlapping display). The Hit rate [Hit rate = Hits/(Hits + Misses)] and the False-Alarm (FA) rate [FA rate = FA/(FA + Correct-Rejections)] were then calculated separately for each participant for the 60% and 80% overlap displays within each sound condition (i.e., imagine damped, imagine ramped, no sound). From the Hit rate and FA rates, each participant’s perceptual sensitivity [*d*′ = Φ^−1^ (Hit rate) −Φ^−1^ (FA rate)] and response bias [*c* = −(Φ^−1^ (Hit rate) + Φ^−1^ (FA rate))/2] were calculated (Fig. 4a,b). Positive perceptual sensitivity index (i.e., *d*′) values indicate better performance in the visual discrimination task, and negative values for the response bias index (i.e., *c*) indicate a bias towards the ‘partially overlapping’ response. Infinite values were corrected using the 1/(2 *N*) rule^37^,^38^. Note that consistent with Grassi and Casco’s^29^ study on the classic cross-bounce illusion, the responses to the 100% overlap displays are encapsulated in this analysis by the false alarm and correct-rejection values. Thus, the Hit-rate and FA rate (and *d*′ and *c* values) cannot be calculated and compared to the partially overlapping display conditions in the same manner because hits, misses, false alarms, and correct rejections would need to be redefined and interpreted with respect to the partially overlapping conditions (e.g., Hits = fully overlapping response given for a fully overlapping display) Moreover, doing so would require collapsing responses across two of the partial overlap conditions (60% and 80%) or leaving the data from one of these conditions out of the analysis, which would undermine a clear interpretation of *d*′ and *c*.

The participants’ sensitivity indices were submitted to a 2 × 3 repeated measures ANOVA, with percent overlap (80% and 60%) and sound condition (imagine damped sound, imagine ramped sound, and no sound) as within-subject factors. This analysis revealed a significant main effect of percent overlap, *F*(1, 23) = 14.71, *p* < 0.001, *η*^2^_G_ = 0.07, but no significant main effect of the sound condition, *F*(2, 46) = 0.22, *p* = 0.80, *η*^2^_G_ = 0.002, nor a significant interaction *F*(2, 46) = 0.49, *p* = 0.61, *η*^2^_G_ < 0.001 was found, suggesting that performance was significantly better in the 60% overlap condition but was uniformly so for all three sound conditions. Planned comparisons also confirmed this, revealing that there were no significant differences in the sensitivity indices for the 60% overlap condition when the damped sound was imagined (*M* = 1.09, *SD* = 1.28), compared to when the ramped sound was imagined (*M* = 0.96, *SD* = 1.28) [*t* (23) = 0.63, *p* = 0.537, *d* = 0.1] or no sound was imagined (*M* = 1.11, *SD* = 1.35)[*t* (23) = −0.12, *p* = 0.90, *d* = −0.02]. There was also no significant difference between when the ramped sound was imagined and no sound was imagined [*t* (23) = −0.80, *p* = 0.43, *d* = −0.11]. The same was true for the 80% overlap condition, with no significant difference between when a damped sound was imagined (*M* = 0.52, *SD* = 0.67) compared to when a ramped sound was imagined (*M* = 0.48, *SD* = 0.71) [*t* (23) = 0.30, *p* = 0.77, *d* = 0.06], or no sound was imagined (*M* = 0.51, *SD* = 0.69) [*t* (23) = 0.11, *p* = 0.91, *d* = 0.01]. There was also no significant difference between when the ramped sound was imagined and no sound was imagined [*t* (23) = −0.21, *p* = 0.84, *d* = −0.04] (Fig. 4a).

The participants’ response bias indices were also subjected to a two-way repeated measures ANOVA, with sound condition (imagine damped sound, imagine ramped sound, and no sound) and percent overlap (80% and 60%) as within subject factors. This analysis revealed a significant main effect of percent overlap, *F*(1, 23) = 14.69, *p* < 0.001, *η*^2^_G_ = 0.013 but no significant main effect of the sound condition, *F*(2, 46) = 0.12, *p* = 0.89, *η*^2^_G_ = 0.001, nor a significant interaction, *F*(2, 46) = 0.49, *p* = 0.62, *η*^2^_G_ < 0.001. Planned comparisons confirmed this, revealing there were no significant differences in the response bias indices for the 60% overlap condition when the damped sound was imagined (*M* = −0.48, *SD* = 1.08) compared to when the ramped sound was imagined (*M* = −0.41, *SD* = 1.16) [*t* (23) = −0.32, *p* = 0.75, *d* = −0.06] or when no sound was imagined (*M* = −0.54, *SD* = 1.18)[*t* (23) = 0.36, *p* = 0.72, *d* = 0.05]. There was also no significant difference in the response bias indices when the ramped sound was imagined compared to when no sound was imagined [*t* (23) = 0.69, *p* = 0.49, *d* = 0.11]. The same was true for the 80% overlap condition, with no significant difference between when a damped sound was imagined (*M* = −0.19, *SD* = 1.24) compared to when a ramped sound was imagined (*M* = −0.17, *SD* = 1.30)[*t* (23) = −0.09, *p* = 0.93, *d* = −0.02] or no sound was imagined (*M* = −0.24, *SD* = 1.33) [*t* (23) = 0.32, *p* = 0.75, *d* = 0.05] (Fig. 4b).

The vividness ratings from the participants in Experiment 1a and Experiment 2 were compared using a Mann-Whitney *U*-test, which revealed that there was no significant difference between the vividness of the participants’ auditory imagery in Experiment 1a (*Mdn* = 7, *IQR* = 2) compared to Experiment 2 (*Mdn* = *7, IQR* = 3.5), *U = *325*, p* = 0.44, *r* = 0.02. This result suggests that there were no differences in the participants’ ability to imagine the sounds that could explain the lack of differences in attention and response bias found for imagined sounds in Experiment 2. The participants’ vividness ratings were neither correlated with the mean difference between ramped and damped conditions (across overlap conditions) for the sensitivity index *d*′ (*r*_s_(22) = 0.13, *p* = 0.55) nor for with response bias index *c (r*_s_(22) = −0.06, *p* = 0.79) in Experiment 2. Additionally, it is worth noting that the majority of the participants reported that they were able to imagine the auditory stimuli with some degree of vividness (i.e., all except one of the forty-eight participants indicated that they were able to imagine the auditory stimuli with vividness ratings greater than one).

## Discussion

The results from this study demonstrate that the content of what one imagines hearing in one’s mind changes what one sees in the external world. Specifically, we have found that while the imagination of a damped sound leads to an increase in the bouncing percept compared to when no sound is imagined, imagining a ramped sound (the same sound as the damped sound, only played backwards) did not. This finding is consistent with the so called ‘unity assumption’ principle of multisensory integration, which states that only meaningful combinations of cross-modal stimuli are preferentially integrated^26^,^27^,^28^. Here, only the imagined damped sound is ecologically relevant, i.e., has acoustic properties typical of colliding objects, and is therefore integrated with the ambiguous visual stimulus in a manner that changes visual perception. This is consistent with results obtained with real auditory stimuli presented here and in previous studies on audiovisual integration^25^,^26^,^27^,^39^.

Furthermore, we found compelling evidence that the present illusory effect represents a genuine perceptual phenomenon. In addition to the main result of the significant difference in reported bounce-percepts between the damped versus no-sound and ramped imagery conditions, this conclusion is based on four main findings: First, we found that the increased proportion of perceived bounce for imagined and perceived damped sounds in Experiments 1a and 1b scaled as a function of the percentage that the discs overlapped in the imagery and real perceptual conditions, respectively. If the participants were responding on the basis of a response bias heuristic such as ‘if I imagine (or hear) the damped sound, I should press bounce,’ their response bias would be betrayed by responding ‘bounce’ with approximately equal frequency in all three overlap conditions. Instead, we observed that extent that the discs overlapped decreased the probability of a bounce percept for damped sound conditions in the same manner it did for the ramped and no-sound conditions, suggesting that the participants were responding on the basis of their genuine perception in all three sound conditions and that the increased probability of a bounce percept observe for damped sounds cannot merely be explained by response bias. Second, in Experiment 2, we found that there were no differences between imagining a damped vs. ramped auditory stimulus at the moment the disks met in terms of the ability to perceive disc overlap (as indicated by the sensitivity index *d*′). This suggests that the two imagery conditions are well matched in terms of demands on central attentional processes. Third, in Experiment 2, we observed no difference in response tendencies (as indicated by the response bias index *c*) across imagery conditions, confirming that the results cannot be explained by a systematic response strategy. Fourth, we found a significant positive relationship between the vividness with which one was able to imagine the sounds during the experiment and the overall strength of the cross-bounce illusion. Taken together, these findings confirm that the increased perception of the bounce-percept in Experiment 1a reflects a genuine change in perception related to imagining the damped sound that cannot be explained by differences in attention or response bias.

The results from Experiments 1a and 1b also provide support for the notion that auditory imagery and auditory perception involve overlapping representations and that imagined auditory stimuli can integrate with visual stimuli to change visual perception. The findings from the experiments presented here are also consistent with our previous study demonstrating that imagining an ecological collision-like auditory stimulus at the moment the discs met, but not before or after, led to an increased bounce-percept^20^, as in the classical illusion with real auditory stimuli^20^,^22^,^23^,^24^. However, although this previous effect nicely demonstrated the temporal specificity of the imagery-induced cross-bounce illusion—in line with the temporal congruency principle of multisensory integration^19^,^40^ ‒the unity assumption principle had not been directly tested. Demonstrating that the illusory increase in the perception that the discs bounce conforms to both the temporal congruency and unity assumption principles provides strong evidence that multisensory integration of real and imagined sensory signals is the underlying mechanism for this change in perception. In addition to this conceptual advance, the present study was also designed to include better controls for attention and post-perceptual effects than our previous study^20^. Berger and Ehrsson^20^ did include a control experiment in which a finger lift was imagined at the moment of coincidence and did not significantly increase the bounce-percept; however, because a finger lift involved directing attention to a different sensory modality, and to the action domain, whether there were unaccounted-for attentional differences between these stimuli could not be fully ruled out. The present design is superior in this respect as we compared imaging two very similar sounds in otherwise equivalent conditions. In sum, the findings presented here rule out alternative explanations for the imagery-induced cross-bounce illusion, and importantly, demonstrate that the *content* of our auditory imagery, not the mere act of imagining an auditory stimulus *per se,* is what changes what we see. This finding provides strong evidence in favor of a multisensory integration account of imagery-induced multisensory illusions. Future neuroimaging experiments may serve to investigate whether the imagery-induced cross-bounce illusion also involves the same multisensory areas involved in integrating audiovisual stimuli in the classic cross-bounce illusion.

## Additional Information

**How to cite this article**: Berger, C. C. and Ehrsson, H. H. The Content of Imagined Sounds Changes Visual Motion Perception in the Cross-Bounce Illusion. *Sci. Rep.* **7**, 40123; doi: 10.1038/srep40123 (2017).

**Publisher's note:** Springer Nature remains neutral with regard to jurisdictional claims in published maps and institutional affiliations.

## Supplementary Material

Supplementary Information

Supplementary Media Video
