# Supplementary material for: The Content of Imagined Sounds Changes Visual Motion Perception in the Cross-Bounce Illusion
Source: Sci Rep. 2017 Jan 10;7:40123. doi: 10.1038/srep40123 (PMC5223176; doi:10.1038/srep40123)
Supplement: Supplementary Information [file srep40123-s1.doc]

**Supplemental Information for:**

**The Content of Imagined Sounds Changes Visual Motion Perception in the Cross-Bounce Illusion**

Christopher C. Berger and H. Henrik Ehrsson

**Inventory of Supplemental Information:**

1. **Supplemental Media**
   1. **Video File:** Examples of Auditory Stimuli Followed by Examples of Stimulus Combinations Containing Imagined/Heard Ramped and Damped Sounds
      1. ***Auditory Stimuli***
         1. Damped sound (played 2x)
         2. Ramped sound (played 2x)
      2. ***Stimulus Combinations for Experiment 1A***
         1. Imagined Damped Sound; 100% Overlap Trial
         2. Imagined Damped Sound; 80% Overlap Trial
         3. Imagined Damped Sound; 60% Overlap Trial
         4. Imagined Ramped Sound; 100% Overlap Trial
         5. Imagined Ramped Sound; 80% Overlap Trial
         6. Imagined Ramped Sound; 60% Overlap Trial
      3. ***Stimulus Combinations for Experiment 1B***
         1. Damped Sound; 100% Overlap Trial
         2. Damped Sound; 80% Overlap Trial
         3. Damped Sound; 60% Overlap Trial
         4. Ramped Sound; 100% Overlap Trial
         5. Ramped Sound; 80% Overlap Trial
         6. Ramped Sound; 60% Overlap Trial
